# Cerebral Sinovenous Thrombosis in a Child with Idiopathic Nephrotic Syndrome

**DOI:** 10.1155/2011/724950

**Published:** 2011-11-30

**Authors:** L. Ghedira Besbes, S. Haddad, A. Gabsi, M. Hassine, Ch. Ben Meriem, M. N. Guediche

**Affiliations:** ^1^Pediatric Department, Fattouma Bourguiba Hospital, Monastir 5000, Tunisia; ^2^Hematology Laboratory, Fattouma Bourguiba Hospital, Monastir 5000, Tunisia

## Abstract

Nephrotic syndrome (NS) is a renal disorder characterized by heavy proteinuria, hypoalbuninemia, edema and hypercholesterolemia. Nephrotic syndrome in children is known to be associated with an hypercoagulable state and thromboembolic complications. However cerebral sinovenous thrombosis (CSVT) is very rare. Here we report a seven-year-old child with steroid-dependent idopathic nephrotic syndrome resulting from a minimal change disease, developed multiple cerebral sinovenous thrombosis, presenting with headache, left sixth nerve palsy, and papilledema. The diagnosis of CSVT was established by cranial computed tomography, magnetic resonance imaging, and magnetic resonance angiography. He gradually recovered after anticoagulant therapy. CSVT is very rare in nephrotic children. The diagnosis of CSVT should be considered in any patient with nephrotic syndrome who develops neurologic symptoms. This report highlights the importance of suspecting and recognizing this potentially life threatening complication and initiating early treatment.

## 1. Introduction

Nephrotic syndrome (NS) is a renal disorder characterized by heavy proteinuria, hypoalbuminemia, edema, and hypercholesterolemia. The reported annual incidence of nephrotic syndrome is between two and seven per 100 000 children aged 1 to 18 years [1].

Children with NS are at risk for venous and arterial thrombosis, uncommon but serious complications of the nephrotic syndrome. Multiple factors contribute to the hypercoagulable state in nephrotic children. The reported incidence of thromboembolic complications in nephrotic children is relatively high, ranging from 1.8% to 5.3% [2].

Cerebral sinovenous thrombosis (CSVT) is very rare and serious, with only few isolated reports in the literature. It may have a nonspecific and variable presentation, and the diagnosis can be difficult without appropriate imaging. CSVT may carry increased morbidity, and its treatment consists mainly of anticoagulant therapy [3].

This report describes a seven-year-old boy with steroid-dependant nephrotic syndrome resulting from a minimal-change nephrotic syndrome, which developed into multiple cerebral sinovenous thrombosis.

## 2. Case Report

A previously healthy boy developed idiopathic nephrotic syndrome at seven years of age. He was initially treated according to the standard protocol of the French Society of Pediatric Nephrology [4] and was steroid sensitive, with complete remission occuring on day ten of treatment with 60 mg/m² per day. Four months later, while the patient was on 15 mg prednisone every other day, he was hospitalized because he developed a first relapse.

Physical examination revealed generalized edema with blood pressure of 110/60 mm Hg and increased body weight of 11 kg over basal conditions. He displayed normal vital signs. He had severe edemas in the face, lower extremities, and abdomen with ascites and bilateral scrotal edema. The rest of his examination was normal.

Laboratory findings included urinary protein excretion >120 mg/kg/day, total serum protein 35 g/L, serum albumin 6.3 g/L, and hypercholesterolemia 17.2 *μ*mol/L, and his electrolyte and renal function were normal. He had normal blood count (hemoglobin 13.6 g/dL, white blood cell count 11500 cells/*μ*L, platelet count 283000/*μ*mol). Serum complements C3 and C4 were normal. The patient was treated by prednisone 60 mg/m² every day. Since he has very low serum albumin levels, he was treated with infusion of human albumin 20% solution (1 g/kg) and diuretic use (furosemide 1.5 mg/kg), both administered in two separate doses; this treatment was repeated 3 days later.

**Figure 1:**
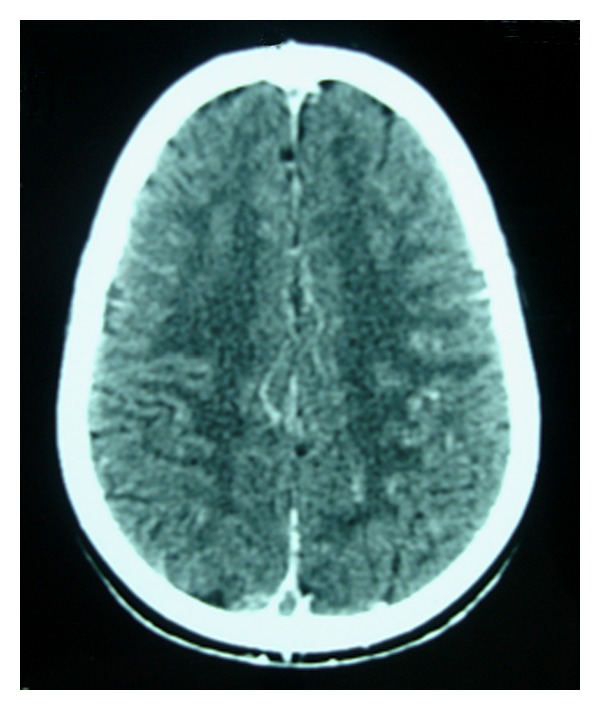
A noncontrast CT scan of brain in axial section showing hyperdense changes (the empty delta sign) consistent with sinovenous thrombosis of *t* posterior part of the sagittal sinus.

On day six of his hospitalization and while on prednisone 60 mg/m²/day, he developed severe headache with sudden onset of convergent strabismus of his left eye without deterioration of his sensorium. The patient had no fever, and blood pressure was normal (95/60 mm Hg), when he was examined in the ophthalmology department, his visual acuity in both eyes was 10/10, both pupils reacted well to light, and the anterior segment was normal. However there was convergent strabismus of the left eye with paralysis of lateral movements of the left eye, and there was a papilledema. To rule out causes of intracranial hypertension, a computed tomography (CT) scan of the brain was performed, and this revealed an extensive sinovenous thrombosis of the superior sagittal sinus (“empty delta” sign), the right transverse sinus, and the right sigmoid sinus (Figure 1). There were no infarcts or hemorrhages. He was diagnosed as having cerebral sinovenous thrombosis (CSVT) secondary to his nephrotic syndrome.

A renal ultrasonogram with the Doppler flow revealed normal-sized kidneys with normal flow in the renal veins.

A coagulation profile showed prothrombin time to be 93% (Ri (reference intervals); 70–100%); activated cephalin time 26 (“normal: 30”), high fibrinogen level 4.47 g/l (Ri: 2–4 g/L), and platelet count 288000/*μ*L.

A thrombophilia screen was done which showed a reduced antithrombin III 61% (Ri: 80–120%), high serum protein C 150% (Ri: 70–120%), and normal serum protein S 81% (Ri: 65–140%) levels. Resistance to activated Protein C was negative. Antiphospholipids and anticardiolipin antibodies were negative. Treatment of cerebral venous thrombosis was started immediately with unfractionated heparin for 10 days that was later changed to subcutaneous low-molecular-weight heparin (LMWH) for two months. The dose of heparin was adjusted according to anti-XA antibody levels (therapeutic range: 0.5–1 unit/mL).

**Figure 2:**
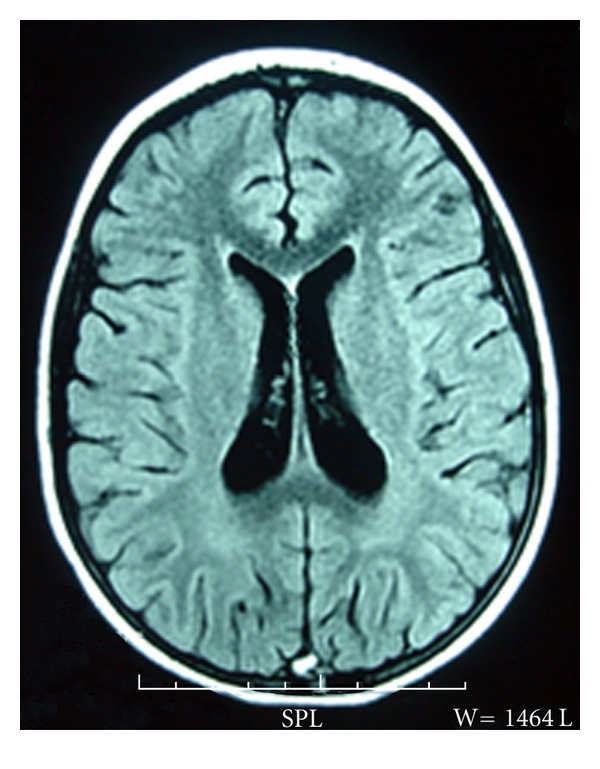
MRI of the brain (FLAIR) showed a high signal in the posterior part of the sagittal sinus.

**Figure 3:**
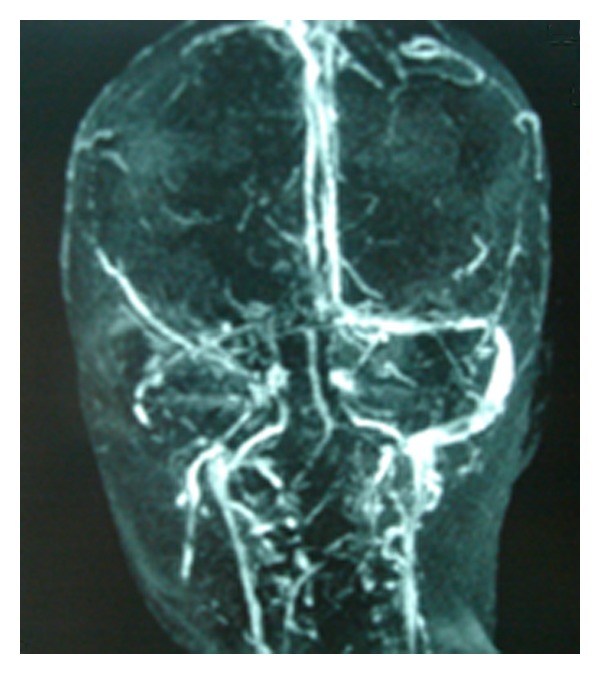
Three-dimensional phase-contrast MRA showed extensive sinovenous thrombosis, involving the superior sagittal sinus, the right sigmoid sinus and the lateral sinus.

Seven days after the administration of heparin treatment, a magnetic resonance imaging (MRI) of the brain and cerebral magnetic resonance angiography (MRA) showed thrombosis of the superior sagittal sinus, the right sigmoid sinus, the right lateral sinus, and the left temporoparietal cortical vein (Figures 2 and 3).

The nephrotic state was treated with prednisone (60 mg/m²/day), and remission of the nephrotic syndrome occurred two weeks after the institution of prednisone.

The child made a complete neurologic recovery (conjugate eye movements appeared to be normal) within one month of treatment.

At the end of the first month of anticoagulant therapy, a cranial MRA was performed and did not show any thrombi in dural sinuses.

After two months of LMWH, treatment was switched over to vitamin K antagonists (Acenocoumarol), and he was being monitored with regular international normalized ratio (INR).

During the six months following the thrombosis complication, the child has had frequent relapses of his nephrotic syndrome; then a percutaneous renal biopsy, ultrasound guided, was performed. The result of renal biopsy was compatible with minimal-change nephrotic syndrome. Anticoagulation medication was continued during one year after the thrombosis and then stopped.

The child remains steroid dependent on low-dose prednisone alternate days, without recurrence of thrombosis and without relapses during the last year.

## 3. Discussion

Cerebral sinovenous thrombosis in children is a serious but uncommon diagnosis that can be associated with several underlying systemic conditions. The current incidence of CSVT in children based on the Canadian registry is 0.67/100.000 [5]. Thromboembolic events are know to complicate nephrotic syndrome. The incidence of thromboembolic complications in nephrotic children is close to 2% with a higher incidence in steroid-resistant NS (3.8%) than steroid-responsive NS (1.5%) [6].

Involvement of cerebral vessels has been rarely reported in nephrotic children [6, 7]. Sinovenous thrombosis is probably less recognized or remains underreported in children with nephrotic syndrome [7].

A recent review of the literature by Fluss et al. of all cases documented of CSVT in nephrotic children has revealed twenty-one cases from 1980 to 2005 [8]. Since this review, eight other cases have been reported: two cases in 2007 [9, 10], one case in 2008 [11], and five cases  in 2010 [12]. With our case, 35 cases of CSVT in nephrotic children are reported.

Clinical presentation of CSVT in children is extremely variable and nonspecific. The diagnosis of CSVT should be considered in any patient with nephrotic syndrome who develops neurologic symptoms: these symptoms include focal or generalized seizures; signs of raised intracranial pressure including headache, vomiting, lethargy, irritability; a decreased level of consciousness, focal neurological deficits such hemiparesis, cranial nerve palsies, and papilledema [8, 13].

In the presence of the appropriate clinical history, brain CT is usually the first investigation performed. In most cases, as the one presented here, the diagnosis of cerebral sinovenous thrombosis can be made on the basis of CT findings, it demonstrates direct signs of thrombosis (cord sign, dense triangle sign, or empty delta sign), edema, cerebral infarction, or parenchymal haemorrhage. However, conventional CT can miss the presence of CSVT in up to 40% of children and underestimate both the extent of the thrombus and the presence of venous infarcts [5].

Brain magnetic resonance imaging (MRI) and MR venography are considered to be a superior modality for diagnosis and follow-up of CSVT. MRI depicts direct signs of thrombosis of one or more venous sinuses, thrombosis of cortical veins, and focal parenchymal lesion. MRV confirms the thrombosis of cerebral sinovenous by demonstrating a lack of flow in the cerebral veins [14]. The most frequently involved sinuses were the superior sagittal sinus followed by the straight sinus and the transverse sinuses [8, 14]. Deep venous system involvement was rarely reported [8, 14].

The relation of NS with hypercoagulability and thromboembolic complications is well known. Hypercoagulation in NS is the consequence of an imbalance between the clotting activator system and the inhibitor system [15, 16]. These include increased procoagulatory activity (fibrinogen, factors V and VIII), urinary loss of anticoagulants (antithrombin III, protein C, and protein S), altered fibrinolytic system, thrombocytosis, and enhanced platelet activation and aggregability [15].

Other factors that increase thrombotic risk in nephrotic children include hyperlipidemia, haemoconcentration, hypoalbuminemia (less than 2.5 g/dL) high rate of protein excretion (more than 10 g/day), low antithrombin III levels, diuretic use, corticosteroid treatment, and indwelling catheters [17].

The risk of thrombosis is higher at the onset of the disease or during a relapse when AT III levels and albumin levels were decreased. Hypoalbuminaemia not only reflects intravascular volume depletion, causing increased blood viscosity, but may also be directly related to platelet hyperaggregability and alteration in the fibrinolytic system [15].

Even though nephrotic syndrome represents an acquired disease that predisposes to thrombosis, some authors have suggested that inherited thrombophilia could increase the risk of thrombosis in nephrotic children and these children must be controlled for hereditary thrombotic disorders [18], especially FV Leiden mutation because NS patients with FV Leiden heterozygous mutation may be at increased risk for developing SVT at early phases of the disease [9]. Balci et al. have reported a 5-year-old boy with FV-Leiden, heterozygous for G1691 A mutation, and NS presenting with cerebral SVT as the initial symptomatology [9].

Acute anticoagulant of choice is heparin. Authors [19, 20] prefer to use unfractionated heparin (UHF) acutely, as the effects of heparin can be reversed if intracranial haemorrhage occurs. Treatment with UHF for 5 to 7 days is often followed by chronic anticoagulation with LMWH or vitamin K antagonists (coumadin) for 3 to 6 months [19, 20].

Adjunction of fresh frozen plasma could be necessary, because efficient heparin therapy requires adequate levels of antithrombin III [10].

Anticoagulation should be carefully monitored, with activated partial thromboplastin time (APTT) for unfractionated heparin, anti-Xa for LMWM, or international normalized ratio for coumadin, to achieve adequate levels for efficacy while preventing overdosage [20]. Anticoagulation therapy with heparin is probably safe and beneficial for children with CSVT with no bleeding complications reported [3].

There are no randomized data on thrombolysis in children [21]. However this treatment has been used with success in isolated cases of CSVT in nephrotic children [21]. Xia et al. have reported five cases of intracranial venous thrombosis in nephrotic children treated with the combined therapy of urokinase, LMWH, and dipyridamole with good results (complete recanalisation of the cerebral venous sinus) and without relapse in follow-up [12].

Prophylactic anticoagulation in children with steroid-resistant or frequent-relapsing NS has been suggested by some authors, owing to presumed greater risk of thrombosis [22]. Other authors have suggested selection for prothrombotic risk. Different biological markers have been suggested, such as hypoalbuminemia (lower than 20 g/L), high fibrinogen levels (above 6 g/L), and antithrombin III deficiency (lower than 70%) [23]. The use of low-dose aspirin (2–5 mg/kg/per dose) has also been considered [23].

The long-term neurologic outcome of sinovenous thrombosis in nephrotic children was good in most cases reported, and no recurrence of thrombotic event was reported [8].

In summary, children with nephrotic syndrome are at risk of thromboembolic complications including CSVT. The present case report highlights the importance of having a high index of suspicion for cerebral sinovenous thrombosis in cases of nephrotic syndrome with any neurological symptoms. If available, MRI coupled with MRV is the imaging modality of choice. Early recognition, immediate anticoagulation therapy, and control of nephrotic syndrome are essential measures to ensure a good prognosis.
